# Techno-Functional and In Vitro Digestibility Properties of Gluten-Free Cookies Made from Raw, Pre-Cooked, and Germinated Chickpea Flours

**DOI:** 10.3390/foods12152829

**Published:** 2023-07-26

**Authors:** Ilgin Dogruer, Basak Coban, Filiz Baser, Sukru Gulec, Banu Ozen

**Affiliations:** 1 Department of Food Engineering, Izmir Institute of Technology, Urla, TR35430 Izmir, Turkey; ilgindogruer@iyte.edu.tr (I.D.); basakcoban@std.iyte.edu.tr (B.C.); filizbaser@iyte.edu.tr (F.B.); sukrugulec@iyte.edu.tr (S.G.); 2 Molecular Nutrition & Human Physiology Laboratory, Izmir Institute of Technology, Urla, TR35430 Izmir, Turkey

**Keywords:** chickpea flour, gluten-free, cookie, digestibility

## Abstract

Chickpea flour, which is produced in various forms, has high protein and fiber content; therefore, it can be a good ingredient for gluten-free cookies. The objective of this study was to investigate and compare the properties of cookies formulated using raw (RCF), cooked (CCF), and germinated (GCF) chickpea flours. The techno-functional properties of these flours were determined, and scanning electron microscope images and mid-infrared spectra were obtained. The rheological properties of cookie doughs were measured along with their mid-infrared spectra. Baked cookies were analyzed for their technological properties as well as their in vitro digestion properties. Sensory analysis was also performed for all the cookies. The most significant difference among the flours was observed in their water retention capacity, and CCF had 119.7% higher water retention capacity compared to RCF. The dough made with CCF had quite different rheological properties from the others. The cookies baked with GCF had the highest baking loss and spread ratio. The CCF-containing cookies had the hardest structure. The cookies made from RCF had a higher resistant starch content followed by the cookies with GCF. All the cookies had similar scores in all aspects tested in the sensory analysis. The use of three different forms of chickpea flour in cookie formulations resulted in products with very different properties; however, their overall acceptability levels were close.

## 1. Introduction

The gluten-free product market, in which bakery products have a sizable share, has a value of around USD 5.6 billion, and it is estimated to reach USD 8.3 billion by 2025 [1]. There are two major driving forces causing this rise in the market value: an increase in the number of patients diagnosed with celiac disease and growing consumer demand for healthy food options. Celiac disease, one of the most prevalent autoimmune diseases, is estimated to affect 0.5–1% of the general population [2]. People who have celiac disease or gluten-related conditions such as wheat allergy and non-celiac gluten sensitivity experience severe health problems even when they consume small quantities of gluten. In addition to individuals who need to follow a gluten-free diet, there has been an increased demand for gluten-free products from those aiming to maintain a healthy eating regimen. However, in general, gluten-free products do not possess the same nutritional, functional, and sensory characteristics as their gluten-containing counterparts. This is because gluten-free products often rely on non-enhanced or fortified starch as their primary ingredients. Celiac patients generally consume gluten-free products with higher glycemic indexes due to the high pre-gelatinized starch content in these items [3].

Replacing gluten in food products is a difficult task due to various issues, including nutritional gaps and challenges related to maintaining the desired texture. Legume flours such as chickpea flour can become good alternatives in gluten-free bakery products due to their compositional properties. Legumes have an important role in the human diet due to their nutritional value, and the chickpea (*Cicer arietinum* L.) holds considerable significance as a prominent member. The chemical compositions of legumes and their flours are balanced in terms of protein, carbohydrates, and dietary fibers, and they have a low glycemic index [4,5]. Chickpea protein is regarded as a protein of high quality, comprising 18 amino acids, 8 of which are essential [6]. The carbohydrate content of chickpeas mostly consists of the monosaccharides ribose, fructose, and glucose. Oligosaccharides, mainly raffinose, ciceritol, and stachyose, are also found in chickpea grains [7,8]. Unsaturated fatty acids, which are mostly linoleic, oleic, and linolenic acids, form a large portion of the fatty acid content of the chickpea grains [4,9]. Moreover, the mineral contents of grains are also important for human health. For chickpeas, Ca, P, Mg, Fe, and K are the minerals that dominate the mineral content [4].

Although chickpea has high nutritional value, it contains some anti-nutritional factors, such as trypsin inhibitors, hemagglutinins, tannins, phytic acid, and saponins. These anti-nutritional factors can be reduced in various ways, including soaking, cooking, and germination [10]. Germination also has other benefits, such as an increase in bioactive components and protein contents [11,12,13]. Studies have shown that germination increases the nutritional value of grains by increasing starch and protein digestibility; γ-aminobutyric acid (GABA) content; the amount of some essential amino acids, such as lysine, leucine, valine, and isoleucine; and the vitamin contents, including riboflavin, niacin, ascorbic acid, and thiamine [14,15]. Increases in free amino acids, sugars, and phenolic compounds are associated with the activation of enzymes such as proteases and amylases [16]. The cooking of legumes prior to obtaining the flour also has the benefits of increased protein digestibility and a reduction in anti-nutritional factors, particularly phytic acid and tannins for chickpeas [13,17]. Different forms of chickpea flour, such as raw, cooked, roasted, fermented, and germinated flours, can be produced, and these forms have different properties [18].

The cookies are especially suitable for the use of gluten-free legume flours since a gluten network, which is very important for the quality of bread, is not that essential [19]. Chickpea flour has been used to replace a portion of wheat flour or has been combined with other gluten-free products in various bakery product formulations [19,20,21,22,23]. It was reported that the partial replacement of rice flour with chickpea flour in a cookie formulation resulted in improvements in attributes related to the consumer acceptability [19]. In another study, a chickpea flour and chestnut flour combination was used for cookie making, and cookies were obtained with better overall acceptability compared to cookies produced with only one of these flours [24]. The replacement of wheat flour with chickpea flour also increased protein and resistant starch contents and the consumer acceptability of cookies while causing no significant changes in their functional properties [25].

Although different forms of chickpea flour are available, a comparison of the properties of cookies formulated with different types of chickpea flour has not been conducted before. Therefore, the aim of this study was to compare the rheological, technological, sensory, and in vitro digestibility properties of the gluten-free cookies made from three types of chickpea flour.

## 2. Materials and Methods

### 2.1. Flours

Raw (RCF) and germinated (GCF) chickpea flours were obtained from Ingro (Karaman, Turkey), and cooked chickpea (CCF) flour was purchased from Naturelka (Aydın, Turkey).

### 2.2. Flour Analyses

#### 2.2.1. Proximate Analysis of Flours

The moisture, fat, protein, and ash contents of the flours were determined according to AOAC methods 925.05, 960.39, 950.48, and 923.03, respectively. The carbohydrate contents were calculated as 100%—total% of other compounds. The crude fiber contents were determined using the AOAC method 14.020. The total phenolic contents of the flours were measured using a spectrophotometric method in the literature, and the results were expressed as mg gallic acid equivalent/L [26]. The values are the averages of 3 measurements.

#### 2.2.2. Physical Properties of Flours

Color measurements for the L*, a*, and b* values were performed with a colorimeter (CR-400 Konica Minolta, Tokyo, Japan). Bulk density was determined following a procedure in the literature [27]. AACC Method 56-11 was used for water retention capacity. Oil absorption capacity, emulsifying (emulsion activity and emulsion stability), and foaming (foaming capacity and foaming stability) properties were determined according to the procedures in the literature [28]. Microscale images of the flours were obtained with a scanning electron microscope (Quanta 250 SEM, USA). Mid-infrared spectroscopic profiles were collected with a Fourier transform infrared (FTIR) spectrometer (Spectrum 100, Perkin Elmer, Waltham, MA, USA) equipped with a DTGS detector. For this purpose, the flours were mixed with KBr (3%), and mid-infrared transmission spectra of the flour–KBr pellets were obtained with 128 scans at 4 cm^−1^ resolution. Air spectra were obtained as the background before each measurement. Five measurements were taken from each flour type.

### 2.3. Cookie Formulations

The cookie formulations included 90 g of either raw, cooked, or germinated chickpea flour. The other ingredients were the same for all the cookies, and they were 1 egg (~60 g), 10 g corn starch (Ingro, Turkey), 60 g margarine (Sana pastry, Turkey), 40 g brown sugar (Takita, Turkey), and 10 g baking powder (Dr. Oetker, Turkey). All the ingredients were blended in a mixer (KitchenAid, Benton Harbor, MI, USA), and 20 g of dough was shaped with a round mold of 5 cm diameter. Then, the cookie doughs were baked in a convection oven (Senox, Turkey) at 175 °C for 10 min. Two batches with 10 cookies/batch were prepared from each type.

### 2.4. Dough Analyses

#### 2.4.1. Rheological Analysis of Doughs

Dough rheology was analyzed with a back extrusion technique using a texture analyzer (TA-XT2i, Stable Microsystems, Godalming, UK) with a 25 mm cylinder probe (P/25), and the parameters were set at a pre-test speed of 2 mm/s, a test speed of 3 mm/s, a post-test speed of 10 mm/s, and a trigger force of 50 g. A standard-size back extrusion cylindrical container (50 mm diameter, capacity of 115 g) and a back extrusion rig (model A/BE) were used. The container was filled with 50 g of cookie dough. A probe penetrated to a depth of 20 mm and then returned to the starting position. The measurement parameters were chosen according to the values given in the database of the equipment, with slight modifications. The cohesiveness and viscosity index values were calculated from the back extrusion profile curve [29]. Two measurements of each cookie batch were conducted.

#### 2.4.2. Fourier Transform Infrared Spectroscopy Analysis of Doughs

Mid-IR spectra of the cookie doughs were obtained with the attenuated total reflectance (ATR) accessory of the FTIR spectrometer (Perkin-Elmer Spectrum 100, Shelton, CT, USA). The doughs were placed on ZnSe crystal, and 96 scans were taken at a 4 cm^−1^ resolution. Air spectra were obtained as the background before each measurement. Five measurements were taken from each batch.

### 2.5. Cookie Analyses

#### 2.5.1. Quality Parameters of Cookies

The diameters and thicknesses of 10 baked cookies per cookie batch were measured with a caliper, and the averages were calculated. The spread ratio of the cookies was calculated by dividing the diameter of the baked cookie by its height [30]. The moisture contents of the cookies were determined by drying approximately 6–9 g of the sample at 105 °C to a constant weight [31]. Baking weight loss (BWL) was determined by measuring the cookie weight before and after baking. The surface color of the cookies was measured by using a colorimeter (CR-400 Konica Minolta, Tokyo, Japan) and a D65 illuminant.

#### 2.5.2. Texture Measurement of Cookies

Textural properties were determined using a texture analyzer (TA-XT2i, Stable Microsystems, UK), and the hardness value was obtained via a 3-point bending test using a 3-point bending rig, with a trigger force of 5 g and a load cell of 5 kg. Also, the pre-test speed of 1.5 mm/s, test speed of 2.0 mm/s, post-test speed of 10 mm/s, and distance of 10 mm were used as measurement parameters, and the distance between the two bottom supports was adjusted to 50 mm. The peak value of the force was recorded as hardness when the cookies were broken into two pieces [32]. Measurements were made with the cookies stored in glass jars 24 h after baking. The measured hardness values were the averages of 5 different cookies per batch.

#### 2.5.3. Nutritional Value Calculation and In Vitro Digestion Analysis of Cookies

Theoretical nutritional values were calculated from the ingredients according to Atwater’s calorie values, as given in Equation (1):

Energy (kcal/100 g) = 9 × fat (%) + 4 × carbohydrate (%) + 4 × protein (%) + 2 × fiber (%), (1)

For in vitro digestion analysis, the nutritionally significant starch components in the gluten-free cookies were determined according to a procedure in the literature [33]. Samples of the gluten-free cookies were mashed in a ceramic mortar. “As eaten” samples were treated for 30 min at 37 °C with pepsin (Sigma EC 3.4.23.1)—guar gum mixture. A 5 mL solution of 0.25 M sodium acetate buffer was then added to the mixture with 5 glass balls. The tubes were incubated at 37 °C in a shaker incubator (ZHWY-200B, Orbital Shaker, Zhicheng, Shanghai, China) after the addition of a 5 mL enzyme combination containing pancreatin (Sigma EC 232-468-9), amyloglucosidase (Sigma EC 3.2.1.3), and invertase (Sigma EC 232-615-7). After 20 and 120 min, 0.1 mL of the samples was collected to determine the amounts of quickly digestible starch (RDS) and slowly digestible starch (SDS), respectively. The samples were denatured for 5 min at 95 °C in a thermal heater after digestion. For free-sugar analysis, the same amount of samples was weighed. Then, 1 M sodium acetate (pH 4.5) (0.25 mL) and distilled water (10 mL) were added to the sample tube. The tubes were placed into a water bath at 90 °C for 30 min. Then, the tubes were removed from the water bath and cooled to 37 °C, and 0.2 mL invertase enzyme was added into each sample. The tubes were again transferred to the water bath at 37 °C for 30 min. After digestion, for the denaturation of the samples, the tubes were placed into a thermal heater at 95 °C for 5 min. For the starch fraction determination step, 50 µL of each sample was placed into a 96-well plate at the appropriate dilutions. One hundred microliters of glucose oxidase/peroxidase reagent containing o-Dianisidine (GAGO20) was added and incubated at 37 °C for 30 min. After the incubation, 100 µL of 6 M H_2_SO_4_ was added into each cell; then, the absorbance of the samples was measured at 540 nm (37 °C). The values were the averages of 6 measurements.

#### 2.5.4. Sensory Analysis

For the sensory analysis, 38 untrained participants (68.4% women; average age: 25.87; age range: 21–56) evaluated 3 types of cookies for their color, flavor, texture, taste, and overall acceptability in a hedonic 1–7 scale (1 is the lowest score, and 7 is the highest score), which is an affective type of sensory test. The sensory panel was conducted in a sensory evaluation laboratory. Individual panel booths that were lit with white light were used during testing. Each sample of the cookies was coded with a distinct number and placed on white plastic plates. The panelists were also given a glass of water to rinse their palates. The sensory study was approved by the Izmir Institute of Technology Scientific Research and Publication Ethics Committee (Number: 19.09.2022-E.96273).

### 2.6. Statistical Analysis

The results were evaluated by analysis of variance (ANOVA) using Minitab (version 19, Minitab Ltd., Coventry, UK) and Tukey’s pairwise comparison test to obtain differences between individual types for different properties (*p* <  0.05).

## 3. Results and Discussion

### 3.1. Flour Properties

The flour properties were determined to explain the differences between the cookies and to see if they could be correlated with the cookie properties. The results of the proximate and functional properties analyses of RCF, CCF, and GCF are listed in Table 1.

The compositional and functional properties of RCF were in agreement with the values given in the literature [34,35]. There were no significant differences between the protein contents among the flour types. Increased protein content with germination was reported in some studies as well as higher moisture and crude fiber contents [35]. However, other studies found no significant variations in the protein contents of germinated flours [12,36]. It was also reported that heat treatment did not affect the protein content of legume flours [36]. GCF used in this study also had the highest moisture content, but the crude fiber content was not significantly different from that of RCF. CCF had the lowest crude fiber among the three types. The crude fiber and ash contents were the highest for RCF. It was reported that soaking during germination can cause a decrease in ash content [37], and this can also be true for flours obtained from the cooked chickpeas. The highest total phenolic content belonged to GCF followed by RCF and CCF. Higher total phenolic content values with germination were also reported in the literature, while a decrease in the phenolic content was observed in the previous studies for the heat-treated legumes [15]. GCF flour had the lowest fat content among the three types. In a study in the literature, it was concluded that the germination causes a decrease in the fat content due to the hydrolysis of the lipids for the seed development [17].

As far as the physical properties of the flours were concerned, CCF (0.70 g/mL) and GCF (0.74 g/mL) had higher bulk density compared to RCF (0.1 g/mL). It has been reported that the bulk densities of several chickpea cultivars have a variation from 0.536 g/mL to 0.571 g/mL [38], which are lower than those of the current study. Color measurements indicated that RCF had the highest L* value with the brightest color among the three types. Only CCF had a positive a* value, which was related to the red color component and was significantly different from the others. A positive b* value represents the yellow color component, and there were no significant differences between the b* values of RCF and GCF, while CCF had a higher b* value, which could be due to the applied heat treatment.

Among the functional properties, the most significant difference was observed in the water retention capacity of the flours; CCF had the highest average value (300.76%), and RCF had the lowest (137.26%). Therefore, CCF had about 2.2 and 1.73 times higher water retention capacity than RCF and GCF, respectively. Water retention capacity is an important techno-functional property for baking applications and can be related to the rheological properties of the dough. In the previous studies, higher oil absorption and water absorption capacities were recorded for the flours from the germinated and the cooked chickpeas [35,39], and this was also true for this study. Increased water absorption capacity was associated with structural changes in macronutrients such as starch due to more available sites for water interaction [17]. Oil absorption capacity is associated with the mouthfeel and the flavor retention properties, and CCF and GCF had improved oil absorption capacities with respect to RCF, as was observed for the heat-treated and germinated flours in the literature [35,39]. There were no significant differences among all the flour types with regard to the emulsion capacity, emulsion stability, and foam capacity, while the foam stability was significantly higher for RCF, followed by CCF and GCF. Emulsion and foaming activities are mostly related to the ability of proteins to be absorbed in the interfacial area. In addition, no significant differences in emulsion and foaming capacities were observed for the flours from the cooked chickpeas [38].

**Figure 1:**
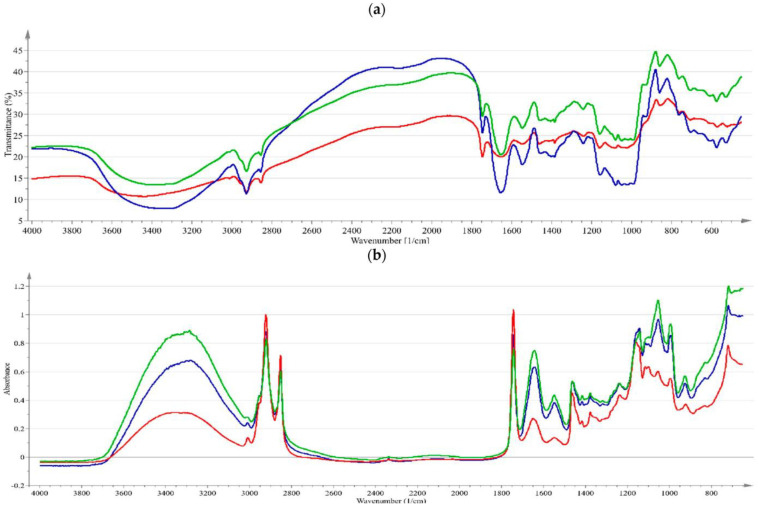
FTIR spectra of (**a**) different types of chickpea flour, (**b**) cookie doughs containing these flours (blue: raw (RCF), red: cooked (CCF), green: germinated (GCF).

**Figure 2:**
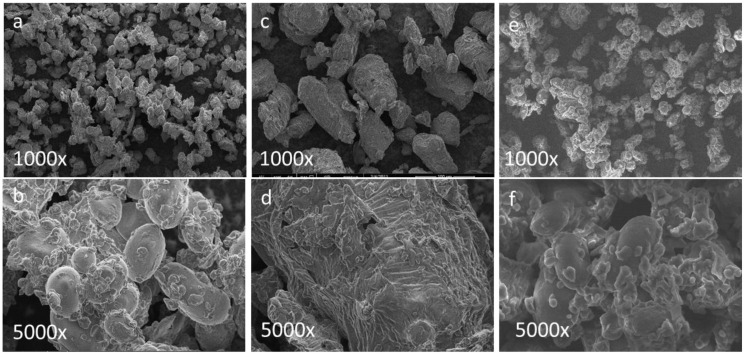
Scanning electron microscope images of (**a**,**b**) raw chickpea flour (RCF), (**c**,**d**) cooked chickpea flour (CCF), (**e**,**f**) germinated chickpea flour (GCF).

The FTIR spectra of the flours were visually inspected to determine the differences in the peaks that were associated with the chemical bonds (Figure 1a). The spectra of RCF and GCF were quite similar in terms of the wavenumber ranges of the peaks although there were differences in the transmittance values, which can be associated with the compositional differences. Previously, it was reported that no significant differences were observed in the FTIR spectra of GCF and RCF flours [35]. However, in the current study, the peaks in the 1500–1400 cm^−1^ and 1100–950 cm^−1^ regions of the CCF spectra had differences in some small peaks with respect to the other types, and these small peaks present in the other two flours with the aforementioned wavenumbers disappeared in the CCF spectra. The 1500–1400 cm^−1^ region is attributed to the peaks due to the -CH_2_ and C-OH deformations for carbohydrates, and the 1100–1000 cm^−1^ area belongs to the fingerprint region and is mostly associated with carbohydrates [40]. The SEM images (Figure 2) also confirm the FTIR results and show that the RCF and GCF microstructures were closer to each other while the images of CCF indicated larger particles, most probably due to water absorption during cooking. The RCF and GCF images also had their differences, and the particles in the RCF images appeared more aggregated. In particular, the micrographs of RCF and CCF clearly showed that starch particles and proteins were located in between them. The CCF and RCF micrographs had similar features to the ones observed in the literature [39].

### 3.2. Cookie Dough Properties

The rheological parameters obtained from the back extrusion measurement of the dough prepared using CCF were significantly different from the doughs made with RCF and GCF (Table 2). The peak force is considered as a firmness value, and a higher value represents a firmer sample. Therefore, the CCF cookie dough had the firmest texture (7.43 ± 1.18 N) among the three types and was significantly different in that respect from GCF and RCF. A high consistency value means a thicker consistency of cookie dough, and the dough prepared with CCF had a consistency value (119.02 ± 8.06 N×s) almost ten times higher than that of the RCF and GCF doughs. The viscosity index value represents the resistance of the dough to movement. The viscosity index and the cohesiveness have negative values, and the absolute values for these measurements are used to evaluate the data. Hence, the cookie dough made with CCF was the most viscous dough, and GCF and RCF had close viscosity index values. A negative peak force was obtained as a cohesiveness value, and the cookie dough made with CCF had the highest cohesiveness. The cookie doughs made with GCF and RCF were less cohesive. These rheological properties of the doughs can be related to the water retention capacity of the flours since strong linear correlations (R^2^: 0.98–0.99) were obtained when the water retention capacity versus all the measured rheological properties was plotted. As all the plots could be constructed with only three points representing each flour, this conclusion should be treated cautiously. CCF, with higher water retention capacity, caused the formation of doughs with higher values of the rheological properties, and this can be attributed to the gelatinization of starch during the cooking of the chickpeas, which is evident from the SEM images and FTIR spectra of the flours. There were some handling problems during the cookie preparations, and the cookie doughs made with RCF and GCF were too sticky to work with. It was very difficult to shape them using a cookie cutter. These observations also confirm the differences among the cookies made with the different flour types.

The FTIR spectra of the doughs prepared from the three types of flour are presented in Figure 1b. As a supporting result for the rheological measurements, CCF had the most distinct spectra among the three flours, and the most important differences were observed in 1200–1000 cm^−1^, corresponding to the carbohydrate region with C-O stretching and C-O-C asymmetric stretching [40]. This is the same region where significant differences were also found in the flour spectra, and it could be associated with the gelatinization of the starch during the pre-cooking of the chickpeas.

### 3.3. Cookie Properties

The baked cookies were analyzed for their physical and functional properties, and Table 3 shows the results of these analyses. All the cookies had significantly different moisture contents and baking losses. The differences in the moisture contents of the cookies could be the result of variations in the moisture contents of the flours as well as the baking losses. The highest baking loss was observed for the cookies containing GCF (15.90%), followed by the RCF (13.40%) and CCF (11.78%) cookies. The spread ratio was identified as a physical property that depends on the flour composition, such as dietary fiber, as well as the rheological properties [19,40]. Again, the spread ratio was the highest for the GCF cookies, while the cookies with RCF and CCF had closer but significantly different spread ratios. The GCF-containing cookies were flat with the largest diameters while the others rose nicely and were thicker. No direct relations were observed between the spread ratio and the rheological measurements of the cookie doughs.

RCF produced the hardest cookies (7.91 N), and the hardness values of the cookies containing CCF (5.03 N) and GCF (4.72 N) were not statistically different from each other (Table 3). The cookies with lower moisture content had higher hardness values, as expected. No correlation was observed between the hardness and any of the measured rheological parameters, including the spread ratio. As opposed to our observations, the differences in the hardness values of cookies made from different types of pinto flour were not statistically significant [36]. These different results between studies can be related to the differences in the ingredients of the cookies.

**Figure 3:**
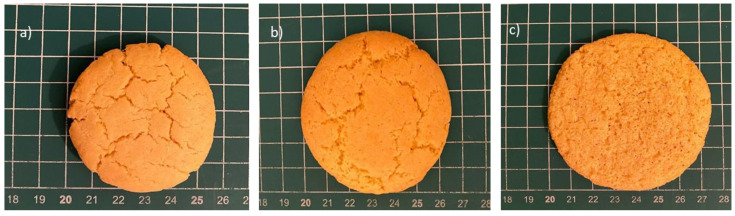
Cookies made from (**a**) cooked chickpea flour (CCF), (**b**) raw chickpea flour (RCF), and (**c**) germinated chickpea flour (GCF).

For the bakery products, color is one of the important features that affect the consumers’ perception of the product. The color analysis indicated that all the cookies had significantly different L* and b* values (Table 3). The cookies made with CCF had the highest L* values, while the cookies containing RCF had the highest b* value. The a* values of the RCF and GCF cookies were very close; however, CCF resulted in the cookies having significantly different a* values. In another study, it was also observed that cookies made from germinated pinto flour had a significantly lower L value compared to raw and cooked pinto flour-containing cookies [36]. The difference in the color properties of germinated flour was associated with an increased occurrence of Maillard browning reactions due to the higher levels of free amino acids and sugars released by enzymes during germination [36]. Figure 3 shows the visual differences among the cookies; the cookies made from CCF had the smallest diameter and a tight form with a cracked surface. The cookies containing RCF also had a cracked surface, but their diameters were larger compared to the CCF cookies. GCF had a porous structure on the surface and had the largest diameter.

The in vitro digestion behaviors of the cookies made with three different chickpea flours were also investigated. Table 4 lists the RDS, SDS, and resistant starch (RS) contents of three cookies made from the three types of chickpea flour. The cookies made from CCF had the highest RDS and SDS contents, while the RS content was lower compared to that of the other cookies. The amounts of starch fractions for the RCF- and GCF-containing cookies were similar to some degree, while the cookies with CCF had quite different profiles. Therefore, it can be concluded that the pre-cooking treatment of chickpea flour caused significant changes in the starch fractions. It has been reported that legumes have poor digestibility because of the inherent physical and structural characteristics of starch and their levels of dietary fiber (especially soluble dietary fiber); therefore, they generally have lower RDS and higher SDS and RS contents when compared to starch from cereal grains [41]. Additionally, starch digestibility can be considerably influenced by the type of ingredients, product composition, and food processing conditions, which have an impact on its metabolic conditions. Consuming foods high in rapidly digestible starch (RDS) increases the risk of obesity and incurable illnesses such as type II diabetes and cardiovascular disease. Rapid starch digestion in the small intestine creates a large and sudden postprandial glycemic peak. Consuming slowly digested starch (SDS), on the other hand, results in a slower release of energy, which aids in preserving glucose homeostasis [42]. The degree of starch digestion in the human small intestine depends on both intrinsic factors, such as granular vs. gelatinized physical structures, and extrinsic factors, such as food viscosity, the surrounding food matrix factors, the amylose/amylopectin ratio, and morphology [43].

The nutritional values of the cookies were calculated theoretically using the compositional data and Atwater’s calorie constants and are listed in Table 4. These cookies made from three different types of chickpea flour provided 484.78–514.72 kcal/100 g, 8.92–9.52% of which came from protein.

The sensory analysis indicated that the overall acceptability of all the cookies was similar (Table 5). In general, relatively low scores were noted for the flavor. Although the cookies had significant differences in the color measurements, the color scores of the sensory analysis were not statistically significant. The same observation was also true for the other measured parameter, texture. According to the statistical results of the sensorial parameters (color, flavor, taste, texture, and overall acceptability), the cookies made from CCF, RCF, and GCF were not significantly different from each other. In another study which aimed to investigate the consumer acceptability of cookies made from raw, cooked, and germinated pinto bean flours, considerable differences were also not observed [36].

While the majority of the crucial parameters that defined the techno-functional and digestibility properties of the cookies were determined, a more comprehensive assessment of these bakery products can be conducted to delve into their sensory attributes and nutritional quality.

## 4. Conclusions

The different types of chickpea flour had their own characteristics, and one of the most significant differences was related to the water retention capacity of the flours. CCF had the highest water retention capacity and had a value that was approximately 119.7% higher than that of RCF. Due to the differences in their characteristics, the doughs and cookies made from these three types of flour also had differences in their properties. The cookies with GCF had the highest baking weight loss and spread ratio, while the RCF-containing cookies were the hardest. Considering the rheological properties of the cookie doughs, the most suitable dough for shaping was the one made with CCF. Our observations during the cookie preparation process also support this information. The cookie doughs made with GCF and RCF were stickier than the dough made with CCF; therefore, the cookies made with CCF had the best handling properties, with nice shape and visual properties. The RCF-containing cookies had the highest proportion of RS, followed by the cookies with GCF. Considering the sensory features, there were no statistically significant differences in the sensory parameters of the cookies. As with the different types of chickpea flour, the cookies made from these flours possessed distinct characteristics, making them suitable for the gluten-free market due to their favorable nutritional attributes. In addition, other types of legume flour, such as carob flour and/or nut flour, can be combined with these different forms of chickpea flour to improve the flavor properties of the cookies.

## Figures and Tables

**Table 1:** Properties of different types of chickpea flour.

Properties	Raw (RCF)	Cooked (CCF)	Germinated (GCF)
Moisture (g/100 g)	4.69 ± 0.12 ^b^	3.30 ± 0.20 ^c^	9.16 ± 0.04 ^a^
Protein (g/100 g)	22.29 ± 1.57 ^a^	22.44 ± 1.68 ^a^	20.76 ± 0.28 ^a^
Fat (g/100 g)	6.38 ± 1.19 ^ab^	10.06 ± 1.27 ^a^	4.66 ± 0.41 ^b^
Carbohydrate (g/100 g)	63.41	63.59	62.61
Total Ash (g/100 g)	3.23 ± 0.05 ^a^	1.95 ± 0.11 ^c^	2.82 ± 0.08 ^b^
Crude Fiber (g/100 g)	3.48 ± 0.19 ^a^	1.96 ± 0.07 ^b^	3.24 ± 0.55 ^ab^
Total Phenol Content (mg GAE/g flour)	0.59 ± 0.20 ^ab^	0.46 ± 0.08 ^b^	0.66 ± 0.04 ^a^
Bulk Density (g/mL)	0.61 ± 0.00 ^c^	0.70 ± 0.00 ^b^	0.74 ± 0.01 ^a^
Water Retention Capacity (%)	137.26 ± 5.24 ^b^	300.76 ± 12.84 ^a^	174.12 ± 12.78 ^b^
Oil Absorption Capacity (g/g)	0.90 ± 0.02 ^b^	1.38 ± 0.13 ^a^	1.01 ± 0.09 ^ab^
Emulsion Activity (%)	46.99 ± 2.81 ^a^	51.00 ± 1.41 ^a^	53.53 ± 0.66 ^a^
Emulsion Stability (%)	96.81 ± 1.37 ^a^	95.15 ± 4.03 ^a^	90.52 ± 2.92 ^a^
Foaming Capacity (%)	17.28 ± 1.41 ^a^	17.29 ± 3.83 ^a^	19.29 ± 4.14 ^a^
Foaming Stability (%)	11.02 ± 1.15 ^a^	8.17 ± 0.24 ^b^	1.84 ± 0.02 ^c^
L*	90.60 ± 1.01 ^a^	88.95 ± 0.91 ^a^	85.80 ± 1.15 ^b^
a*	−5.52 ± 0.60 ^c^	0.91 ± 0.04 ^a^	−3.87 ± 0.20 ^b^
b*	25.20 ± 0.70 ^b^	31.86 ± 0.45 ^a^	24.20 ± 1.57 ^b^

Values are mean ± SD. Means having different letters are significantly different (*p* < 0.05).

**Table 2:** Rheological properties of cookie doughs.

Properties	Raw (RCF)	Cooked (CCF)	Germinated (GCF)
Firmness (N)	4.66 ± 0.63 ^b^	40.23 ± 7.26 ^a^	7.43 ± 1.18 ^b^
Consistency (N × s)	10.885 ± 0.74 ^b^	119.02 ± 8.06 ^a^	18.12 ± 3.70 ^b^
Viscosity Index (N × s)	−4.18 ± 0.62 ^a^	−15.26 ± 4.5 ^b^	−5.40 ± 0.87 ^a^
Cohesiveness (N)	−3.60 ± 0.53 ^a^	−15.19 ± 1.91 ^b^	−4.77 ± 0.81 ^a^

Values are mean ± standard deviation. Means with different letters are significantly different (*p* < 0.05).

**Table 3:** Characteristics of the cookies made from different types of chickpea flour.

Properties	Raw (RCF)	Cooked (CCF)	Germinated (GCF)
Moisture (%)	11.52 ± 0.29 ^a^	9.94 ± 0.71 ^b^	7.46 ± 0.94 ^c^
Baking Weight Loss (%) (BWL)	13.40 ± 0.81 ^b^	11.78 ± 0.73 ^c^	15.90 ± 1.01 ^a^
Spread Ratio	4.53 ± 0.14 ^b^	3.89 ± 0.33 ^c^	7.88 ± 0.65 ^a^
Hardness (N)	7.91 ± 1.05 ^a^	5.03 ± 1.67 ^b^	4.72 ± 1.31 ^b^
L*	68.02 ± 1.75 ^b^	72.71 ± 0.84 ^a^	61.95 ± 2.16 ^c^
a*	2.07 ± 0.45 ^a^	−1.45 ± 0.42 ^b^	1.83 ± 0.95 ^a^
b*	46.27 ± 0.85 ^a^	40.48 ± 0.84 ^b^	39.18 ± 0.99 ^c^

Values are mean ± standard deviation. Means with different letters are significantly different (*p* < 0.05).

**Table 4:** Starch fractions and nutritional values of cookies containing different types of chickpea flour.

	Raw (RCF)	Cooked (CCF)	Germinated (GCF)
Starch fractions *			
Rapidly digestible starch (RDS)	2.58 ± 0.31 ^c^	24.63 ± 0.15 ^a^	7.09 ± 0.52 ^b^
Slowly digestible starch (SDS)	3.45 ± 0.29 ^c^	7.46 ± 1.34 ^a^	5.96 ± 0.16 ^b^
Resistant starch (RS)	38.59 ± 0.25 ^a^	13.10 ± 1.37 ^c^	29.69 ± 0.66 ^b^
Nutritional Values **			
Protein/100 g	12.00	11.48	11.52
Fiber/100 g	1.32	0.73	1.27
Fat/100 g	25.09	26.02	25.16
Carbohydrate/100 g	56.90	58.29	52.43
kcal/100 g	504.05	514.72	484.78
Protein/100 kcal	2.38	2.23	2.38
Fiber/100 kcal	0.26	0.14	0.26
Energy % from protein	9.52	8.92	9.51

* g/100 g dry basis. ** Atwater’s calorie constants. Energy (kcal/100 g) = 4 × (% sugar + % starch + % protein) + 2 × (% dietary fiber) + 9 × (% fat). Values are mean ± SD. Means with different letters are significantly different (*p* < 0.05).

**Table 5:** Sensory evaluation results of cookies containing different types of chickpea flour.

Sensory Properties *	Raw (RCF)	Cooked (CCF)	Germinated (GCF)
Color	5.68 ± 1.09 ^a^	5.32 ± 1.24 ^a^	5.45 ± 1.33 ^a^
Flavor	4.82 ± 1.54 ^a^	4.18 ± 1.52 ^a^	4.76 ± 1.58 ^a^
Texture	5.18 ± 1.54 ^a^	5.16 ± 1.38 ^a^	4.55 ± 1.64 ^a^
Taste	5.08 ± 1.55 ^a^	4.42 ± 1.57 ^a^	4.34 ± 1.46 ^a^
Overall acceptability	5.18 ± 1.29 ^a^	4.72 ± 1.17 ^a^	4.66 ± 1.34 ^a^

* Scale: 1–7. Values are mean ± SD. Means having different letters are significantly different (*p* < 0.05).

## Data Availability

Data will be available on request.
